# A Feasibility Study of Brain‐WISE: A Group‐Based, Person‐Centered Dementia Risk Reduction Program

**DOI:** 10.1002/alz.085559

**Published:** 2025-01-09

**Authors:** Matthew L. Cohen, Kimberly Van Buren, Mindy J Myers, James M Ellison, Christopher R. Martens, Alyssa M Lanzi

**Affiliations:** ^1^ University of Delaware, Newark, DE USA; ^2^ Thomas Jefferson University, Sidney Kimmel Medical College, Philadelphia, PA USA

## Abstract

**Background:**

Reducing risk of dementia requires a person to have accessible information about health and risk factors, person‐centered health goals, and self‐efficacy. Here, we test the feasibility of a new risk reduction program called *Brain Wellness Information, Support, and Empowerment* (Brain‐WISE). Its unique features are that it (1) is aligned with a theoretical model of behavior change, (2) is relatively brief (six 90‐min sessions), (3) is conducted with groups of people who have pre‐existing community; (4) includes individual and group activities and person‐centered health goals; and (5) is conducted with speech‐language therapists.

**Methods:**

We conducted two waves of a single‐arm pilot study with 49 older adults (aged 64‐93) to iteratively refine the program and evaluate its feasibility (adherence, acceptability, and pilot efficacy). Each of the six treatment sessions included a group lecture on brain health topics interspersed with individual and group activities. Brain health topics included (1) normal and abnormal cognitive aging and management of chronic conditions; (2) physical activity; (3) MIND Diet; (4) hearing, cognitive, and social health; (5) preventative use of cognitive aids and strategies; and (6) stress management and sleep. Adherence was measured with attendance. Acceptability was measured with bespoke questionnaires. Patient‐reported outcome measures were administered before and 1 week after treatment to assess changes in intermediate psychological outcomes (e.g., self‐efficacy) and ultimate behavioral outcomes (e.g., physical activity).

**Results:**

Between Waves 1 and 2 we modified lecture content and activity materials, and selected different outcome measures. Attendance in Waves 1 and 2 was 97% and 93.4%, respectively. In each domain of acceptability (lecture content, lecture delivery, and activities), session‐specific mean responses on a 5‐point scale ranged from 4.1 to 4.7. In Waves 1 and 2, 92.3% and 90% of participants agreed or strongly agreed that the program was worthwhile. After Wave 2, pre‐post dementia knowledge increased, MIND diet scores improved, exercise increased, and perceived barriers to lifestyle change decreased. Self‐efficacy did not change.

**Conclusions:**

The Brain‐WISE program is a feasible dementia risk reduction program for community‐dwelling older adults. Based on these findings, a full‐scale RCT to evaluate its efficacy is warranted.

